# Mechanical strain modulates age-related changes in the proliferation and differentiation of mouse adipose-derived stromal cells

**DOI:** 10.1186/1471-2121-11-18

**Published:** 2010-03-10

**Authors:** See-Chang Huang, Tzu-Chin Wu, Hsiao-Chi Yu, Mei-Ru Chen, Chun-Min Liu, Wen-Sheng Chiang, Kurt M Lin

**Affiliations:** 1 Biomedical Engineering Research Laboratories, Industrial Technology Research Institute, Hsinchu, Taiwan; 2 Division of Medical Engineering Research, National Health Research Institutes, Zhunan Town, Miaoli, Taiwan; 3 Department of Biomedical Imaging and Radiological Sciences, National Yang Ming University, Taiwan

## Abstract

**Background:**

Previous studies on the effects of aging in human and mouse mesenchymal stem cells suggest that a decline in the number and differentiation potential of stem cells may contribute to aging and aging-related diseases. In this report, we used stromal cells isolated from adipose tissue (ADSCs) of young (8-10 weeks), adult (5 months), and old (21 months) mice to test the hypothesis that mechanical loading modifies aging-related changes in the self-renewal and osteogenic and adipogenic differentiation potential of these cells.

**Results:**

We show that aging significantly reduced the proliferation and increased the adipogenesis of ADSCs, while the osteogenic potential is not significantly reduced by aging. Mechanical loading (10% cyclic stretching, 0.5 Hz, 48 h) increased the subsequent proliferation of ADSCs from mice of all ages. Although the number of osteogenic colonies with calcium deposition was increased in ADSCs subjected to pre-strain, it resulted from an increase in colony number rather than from an increase in osteogenic potential after strain. Pre-strain significantly reduced the number of oil droplets and the expression of adipogenic marker genes in adult and old ADSCs. Simultaneously subjecting ADSCs to mechanical loading and adipogenic induction resulted in a stronger inhibition of adipogenesis than that caused by pre-strain. The reduction of adipogenesis by mechanical strain was loading-magnitude dependent: loading with 2% strain only resulted in a partial inhibition, and loading with 0.5% strain could not inhibit adipogenesis in ADSCs.

**Conclusions:**

We demonstrate that mechanical stretching counteracts the loss of self-renewal in aging ADSCs by enhancing their proliferation and, at the same time, reduces the heightened adipogenesis of old cells. These findings are important for the further study of stem cell control and treatment for a variety of aging related diseases.

## Background

Recent findings on age-related changes in adult stem cells and stem cell niches suggest that the aging process and aging-related diseases may involve age-dependent stem cell loss, including alterations in their numbers and/or differentiation potential, although many of the details are still not understood [1]. One example of aging-related diseases is osteoporosis in the elderly, in which bone loss and increased bone marrow fat may result from reduced osteogenic potential and a tilted osteogenic/adipogenic balance in bone marrow mesenchymal stem cells (BMMSCs) [2,3]. Another example is obesity, which can be a risk factor for diabetes and cardiovascular diseases and involves excess accumulation of white adipose tissue that is differentiated from MSCs [4]. Most of the current knowledge regarding adult stem cells derives from studies of MSCs isolated from bone marrow. Displaying similar differentiating potentials to BMMSCs, ADSCs possess clear advantage in clinical uses due to easy and repeatable access as well as simple isolation and expansion procedures that promise broad applications for cell therapy and tissue engineering [5,6]. Age-related declines in the lifespan, proliferation, and differentiation capacity of human and mouse BMMSCs have been reported previously [7-12], but most of the mechanisms are still unclear. Aging effects in human and murine ADSCs were only partially explored, and it appears that the osteogenic differentiation capacity of ADSCs is maintained with aging [5,13].

Effects of mechanical force on growth rate, signal transduction, and cell phenotype have been widely documented in a variety of cell types. Mechanical loading was reported to induce osteogenic differentiation [14-17] and smooth muscle cell differentiation [18-20] in BMMSCs, or lead to inhibition of adipogenesis [14] through durable β-catenin activation [21]. In growing mice, exposure to low-magnitude mechanical signals alters the cell fate of BMMSCs by inhibiting adipogenesis [22]. Uniaxial strain inhibited the proliferation of human ADSCs and the expression of early smooth muscle cell markers [23]. In this study, we looked for aging-related differences in ADSCs isolated from young, adult, and old mice. By applying mechanical strain, we tested the hypothesis that mechanical loading counteracts the effects of aging by modulating the self-renewal and differentiation potential of murine ADSCs.

## Results

### Age-related changes in mouse ADSCs

**Figure 1:**
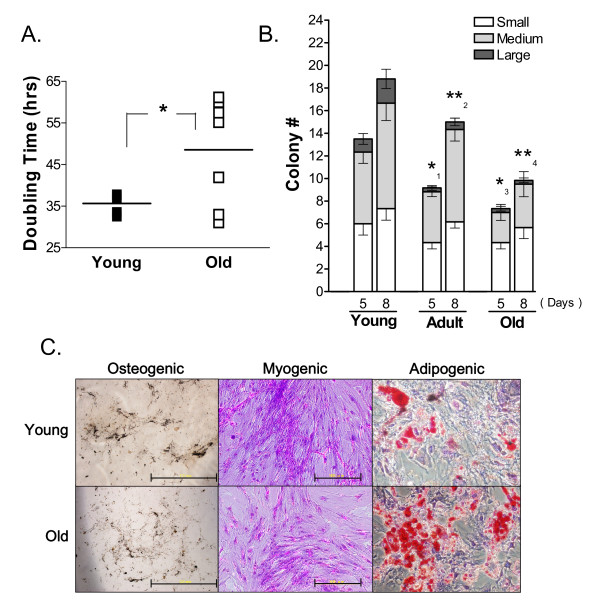
**Renewal and differentiation capacity of ADSCs from old, adult, and young mice**. **A**. Doubling time of P0 ADSCs. *:*P *< 0.04, N = 5 for young ADSCs, N = 7 for old ADSCs. **B**. Number of small (5-10 cells/colony), medium (10-50 cells/colony), and large (> 50 cells/colony) colonies formed after 5 or 8 days of culture after initially plating 5000 young, adult, or old P0 ADSCs. Young ADSCs formed significantly more large colonies than either adult or old ADSCs. *_1_: *P *< 0.04; **_2_: *P *< 0.006 between adult and young ADSCs for the 5- and 8-day colonies; *_3_: *P *< 0.02; **_4_: *P *< 0.004 between old and young ADSCs for the 5- and 8-day colonies. ADSCs from the same mouse were plated in triplicates. N = 8. **C**. Osteogenic (von Kossa staining), myogenic (Liu's staining), and adipogenic (Oil-Red O staining) differentiation in young and old P1 ADSCs responding to differentiating medium after 21 days. The scale bars are 2.0 mm (von Kossa) and 500 μm (Liu's).

To explore the effect of donor age on ADSCs, we isolated ADSCs from a stromal-vascular cell fraction (SVF) derived from the gonadal fat pads of young (8-10 weeks), adult (5 months), and old (21 months) mice. The proliferation rate of passage zero (P0) ADSCs was studied by measuring their average doubling time. The doubling time of ADSCs isolated from young mice was significantly shorter than that of ADSCs from old mice (Figure 1A). Proliferation of ADSCs was also measured by a colony forming assay in which we plated 5000 young, adult, or old P0 ADSCs and counted the colonies formed and the number of cells in each colony after 5 or 8 days of culture. The large colonies (> 50 cells per colony) are of particular importance because they are formed by the most active ADSCs with sustained proliferating ability. This assay demonstrated that young ADSCs formed significantly more large colonies than either adult or old ADSCs (Figure 1B).

To test the differentiation potential of ADSCs, P1 cells were subjected to osteo-, myo-, and adipogenic induction for 21 days followed by von Kossa, Liu's, or Oil-Red O staining (Figure 1C). Compared to young ADSCs, old ADSCs exhibited reduced von Kossa staining and formed fewer multinucleated myotubes, indicating a reduction in calcium deposition and myogenic differentiation. The Oil-Red O staining of old ADSCs was significantly stronger than young ADSCs, indicating that the adipogenic potential was elevated by aging.

**Figure 2:**
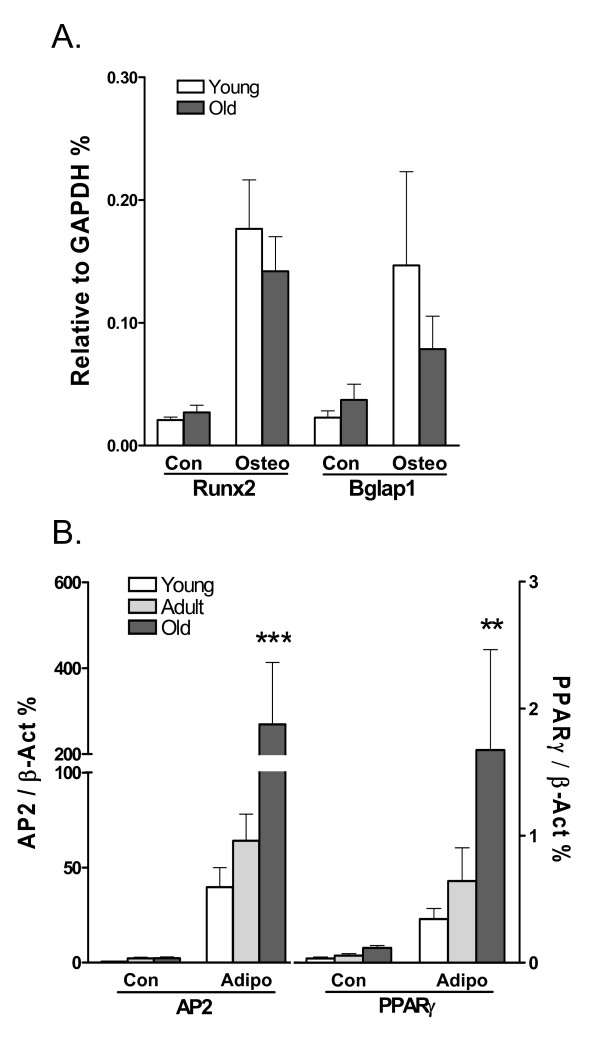
**Expression of osteogenic and adipogenic marker genes in young and old ADSCs**. **A**. Expression of Runx2 and Bglap1 relative to GAPDH in young and old ADSCs after osteogenic induction (Osteo) for 21 days. The marker expression in non-induced cells (Con) is shown. No significant difference was found between young and old ADSCs. N = 6. **B**. AP2 and PPARγ gexpression relative to β-actin expression in young, adult, and old ADSCs subjected to adipogenic induction (Adipo) for 14 days. The non-induced samples were induced for 21 days as in A. ***: *P *< 0.001, **: *P *< 0.01 (N = 3), compared ΔΔCT to young or adult ADSCs.

We measured the expression of bone marker genes, Runx2 and Bglap1, in young and old ADSCs following 21 days of osteogenic induction. Induction with osteogenic medium led to an increase in the expression of Runx2 and Bglap1 in both young and old ADSCs compared to non-induced cells. However, the expression of these bone marker genes, after being normalized to the GAPDH expression, was not significantly different between young and old ADSCs (Figure 2A). This result suggests that fewer von Kossa-stained colonies were formed by old ADSCs likely due to a reduction in the number of colonies, but not a reduction in the osteogenic potential of old ADSCs. In contrast, following adipogenic induction there was an age-dependent increase in the expression of the AP2 and PPARγ genes (Figure 2B), indicating a higher capacity for adipogenesis in old ADSCs.

### Mechanical loading counteracts aging-dependent changes

#### Pre-exposure to mechanical strain increases ADSC self-renewal

**Figure 3:**
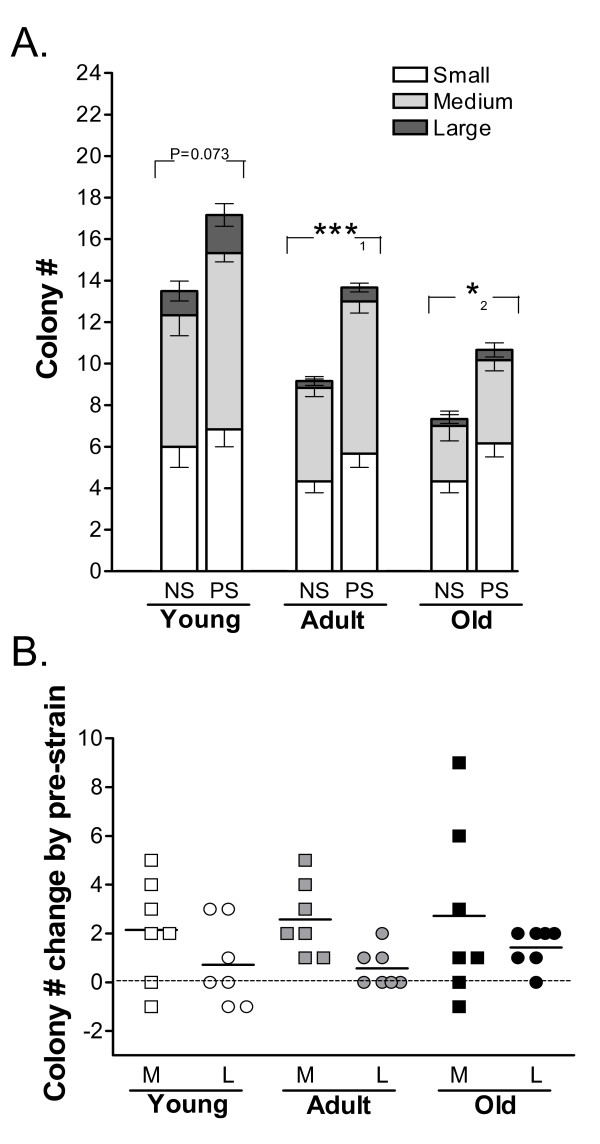
**Mechanical loading improves the colony-forming ability of young, adult, and old ADSCs**. **A**. Number of colonies after 5 day culture of ADSCs, which were pre-exposed to mechanical strain (PS) or as non-strain controls (NS). Colony size was defined as in Figure 1. ***_1_: *P *< 0.001, *_2_: *P *< 0.03. N = 6. **B**. Increased numbers of medium (M) and large colonies (L) due to pre-exposure to mechanical strain in 8-day ADSC cultures. Each point represents the average of triplicate measurements of ADSCs from one mouse. N = 7.

To investigate the effect of mechanical loading, we subjected P2 ADSCs to 48 h of equibiaxial cyclic strain (in a 0.5 Hz sinusoidal waveform with a maximum strain of 10%) and, after a subsequent passage, assessed their colony-forming ability. As shown in Figure 3A and 3B, 48 h of mechanical loading led to a significant increase in the number of colonies formed by ADSCs. This increase in colony number was more pronounced in adult and old ADSCs than in young ADSCs, likely due to the high basal colony-forming ability of unstimulated young ADSCs.

#### Pre-exposure to mechanical strain increases calcium deposition, but not osteogenic potential in ADSC colonies

**Figure 4:**
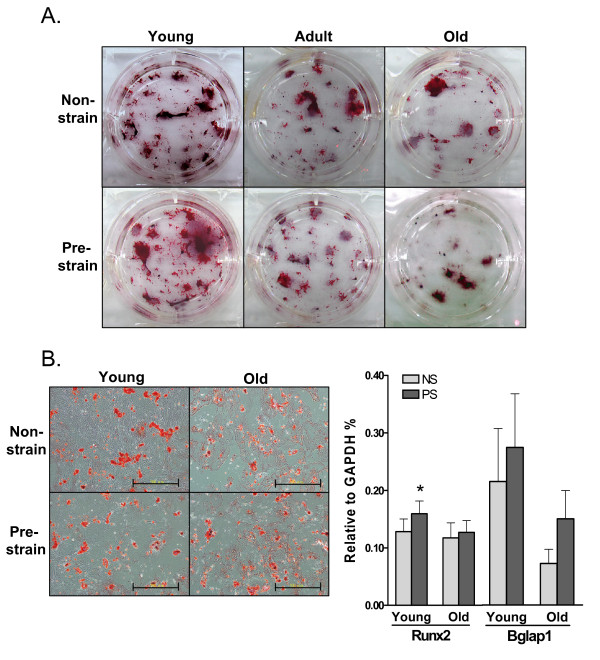
**A. ARS staining for calcium deposition in osteogenically-induced young, adult, and old ADSCs with or without pre-exposure to mechanical strain**. **B**. **Left **Osteogenic colonies from young and old ADSCs with or without pre-strain. Young ADSCs have stronger ARS staining than old cells. No difference in ARS staining caused by pre-strain was found. Scale bars = 500 μm. **Right **The expression of Runx2 and Bglap1 in young and old ADSCs with or without pre-strain. N = 8. *: *P *< 0.05, compared to non-strained young ADSCs.

**Figure 5:**
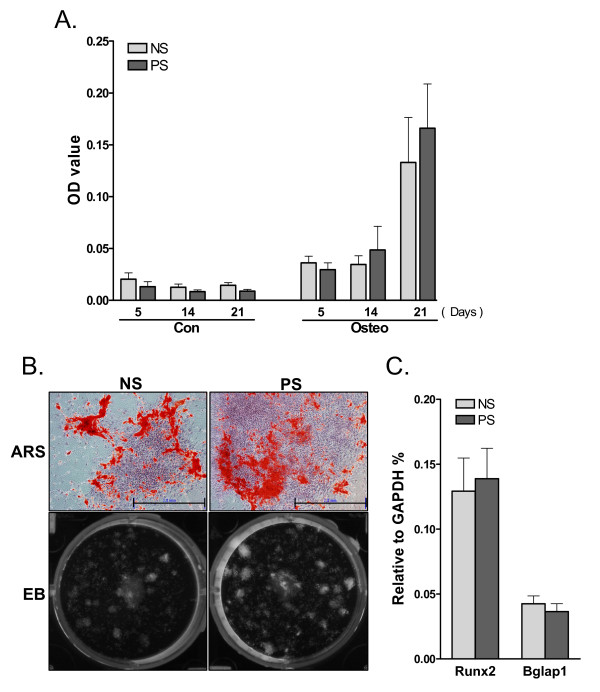
**Pre-strain does not enhance osteogenic differentiation in adult ADSCs**. **A**. The extracted ARS stain normalized to the DNA content of adult ADSCs which were either pre-strained or unstrained, followed by osteogenic induction for 5, 14, or 21 days. A gradual increase in ARS stain was found following osteogenic induction, but no significant difference between the pre-strain and non-strain groups was found. N = 6. **B**. ARS staining of osteogenic colonies (day 14) of adult ADSCs with or without pre-strain. Scale bars = 2.0 mm. Ethidium bromide (EB) staining of the same well is shown below. White pixels in the culture well represent positive EB staining and the pre-strained ADSCs formed more colonies than non-strained ADSCs. **C**. Expression of Runx2 and Bglap1 in pre-strained or non-strained adult ADSCs after 14 days of osteogenesis. No significant difference was found between pre-strained and non-strained samples. N = 6.

To investigate osteogenic differentiation in ADSCs pre-exposed to mechanical strain, the ADSCs were replated following mechanical loading and induced by osteogenic induction medium for various periods of time. As shown in Figure 4A, ADSCs from young, adult, and old animals exhibited an age-related decrease in Alizarin-Red-S (ARS) staining, confirming the result shown in Figure 1C. Pretreatment with mechanical strain appears to result in an increase in ARS staining. However, the detailed analysis shown in Figure 4B demonstrated similar ARS staining density in samples with or without pre-strain. Aging remains the only determinant factor of ARS staining intensity. We also measured the expression of osteogenic marker genes in ADSCs after 21 days of osteogenic induction and found that Runx2 or Bglap1 expression was not significantly altered by pre-strain, except that Runx2 expression was enhanced slightly in pre-strained young ADSCs. These data suggest that pre-strain did not significantly induce osteogenesis of ADSC colonies. We further confirmed this conclusion using quantitative ARS staining after osteogenic induction in adult ADSCs. We quantified the staining results by measuring the absorbance of extracted ARS staining and the fluorescence emitted by ethidium bromide, which stains the DNA and is used as an indirect indication of the cell number contained in each culture well. As shown in Figure 5A, the normalized ARS absorbance gradually increased in osteogenically induced ADSCs, but did not increase in un-induced cells, indicating the validity of our analysis. This experiment also showed that pre-strain treatment failed to result in significant increases in ARS staining. Detailed pictures of ARS colonies and ethidium bromide staining are shown in Figure 5B. The expression of osteogenic marker genes in adult ADSCs, after normalization to GAPDH expression, was also not enhanced by pre-strain (Figure 5C). Therefore, we conclude that pre-strain increases the proliferation of ADSCs, and that the increase in ARS-stained colonies formed by pre-strained ADSCs is due to an increase in the number of colonies formed, but not an increase in the osteogenic potential of the colonies after mechanical loading.

#### Pre-exposure to mechanical strain inhibits adipogenic differentiation in aging ADSCs

**Figure 6:**
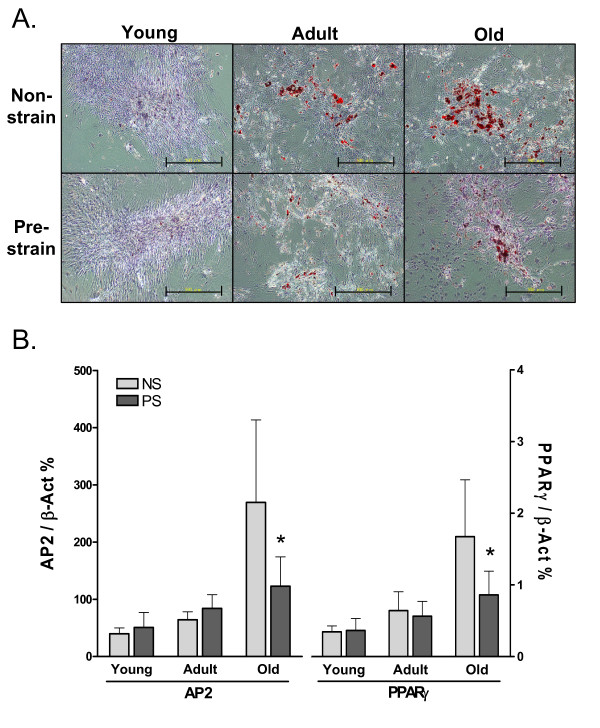
**Aging-related increase in adipogenic capacity of ADSCs and attenuation by pre-exposing to mechanical strain**. **A**. Oil-Red O staining of adipogenic colonies (14 days) formed by young, adult, and old ADSCs with or without pre-strain. Scale bars = 500 μm. **B**. AP2 and PPARγ expression in ADSCs that were with or without pre-strain and were adipogenically induced for 14 days *: *P *< 0.05, compared to non-strained old ADSCs. N = 3.

In contrast to the result in osteogenesis, pre-strain significantly reduced adipogenic differentiation in ADSCs, as shown by the Oil-Red O staining result in Figure 6A. Very few oil droplets were found in young ADSCs after induction, compared to adult and old ADSCs. There were much fewer oil droplets in pre-strained adult and old ADSCs than in non-strained cells. The expression of AP2 and PPARγ in old ADSCs after adipogenic induction for 14 days was significantly reduced by pre-strain, indicating a strong inhibitory effect of mechanical loading on adipogenesis (Figure 6B). The effect of aging and pre-strain on ADSCs is summarized in Table 1.

**Table 1:** Aging related changes in ADSCs and response to mechanical loading.

	Aging	Mechanical Loading
**Self-renewal**	↓	↑
**Osteogenesis**	---	---
**Adipogenesis**	↑	↓

↑ denotes an increase, ↓ denotes a decrease, and --- denotes no change. The change in myogenesis by aging was only partially studied in this study, thus, not reported here.

#### Stronger inhibition of adipogenesis by simultaneous mechanical loading and differentiation induction

**Figure 7:**
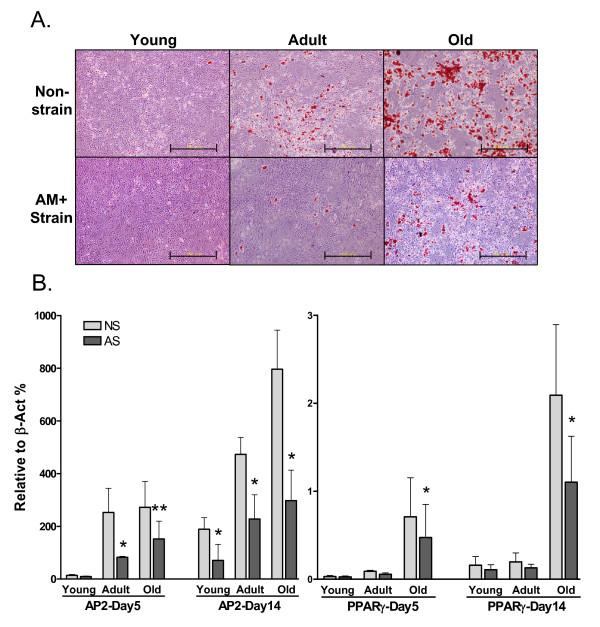
**Strong inhibition of adipogenesis in ADSCs by simultaneously applying mechanical strain and adipogenic induction**. **A**. Oil-Red O staining of ADSCs (AM+Strain) that were strained in adipogenic medium for the first 48 h and then by medium alone for the following 12 days. The non-strain cells were adipogenically induced for 14 days. Scale bars = 500 μm. **B**. AP2 and PPARγ expression in ADSCs (AS) subjected to simultaneous strain for 48 h and adipogenic induction for a total of 5 or 14 days. The non-strained cells (NS) were adipogenically induced for 5 or 14 days **: P < 0.01, *: P < 0.05, N = 3, compared ΔΔCT to NS groups.

**Figure 8:**
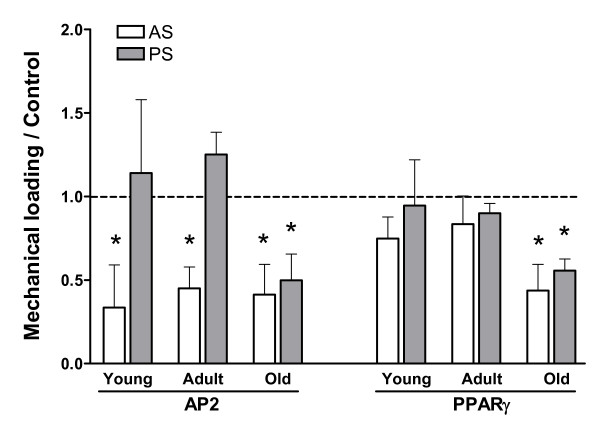
**Comparison of the effect of pre-strain (PS) or simultaneous strain and adipogenic induction (AS)**. The levels of AP2 and PPARγ were normalized to β-actin and compared to the levels in NS groups that were induced for 14 days. Simultaneous strain applied during adipogenic induction was more effective at reducing adipogenic potential in ADSCs. *: P < 0.05, compared to 1. N = 3.

In the pre-strain experiments, ADSCs were passaged after 48 h of mechanical loading and cultured for an additional five days in maintenance medium without mechanical stimulation, followed by differentiation induction. To explore the effect of mechanical loading during differentiation induction, we applied the same pattern of mechanical stretching to ADSCs simultaneously with adipogenic induction. The Oil-Red O staining shown in Figure 7A demonstrates a strong inhibition of adipogenesis in both adult and old ADSCs by a simultaneously applied mechanical loading. A significant reduction in marker expression by strain was observed in adult and old ADSCs under adipogenic induction for only five days (Figure 7B). The differing abilities of the two mechanical loading methods in inhibiting AP2 and PPARγ expression are shown in Figure 8, where simultaneously applied mechanical strain during adipogenic induction significantly reduced AP2 expression in young, adult, and old ADSCs, while pre-exposure to mechanical strain only reduced AP2 and PPARγ expression in old ADSCs. In addition, we also examined the effects of simultaneous mechanical stretching and osteogenic induction in adult ADSCs, and obtained results similar to those of the pre-strain study (data not shown).

**Figure 9:**
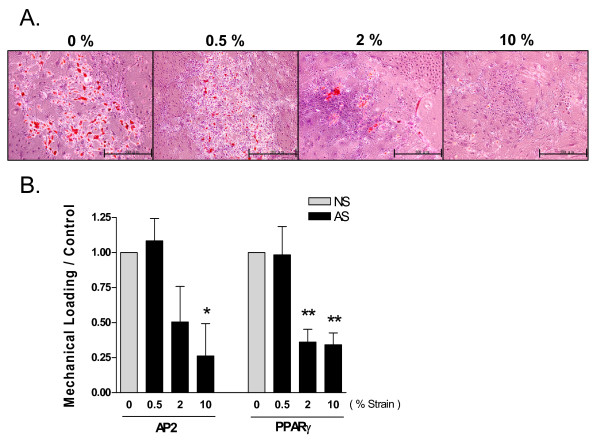
**Strain-does dependent effect on adipogenesis in ADSCs**. **A**. Oil-Red O staining of adult ADSCs that were subjected to AS of various strain magnitudes for the first 48 h and then adipogenic medium alone for the next 8 days. The non-strain cells (0%) were adipogenically induced for 10 days. Scale bars = 500 μm. **B**. AP2 and PPARγ expression in ADSCs subjected to various AS as in A. The result shown was the expression levels normalized to β-actin and to the non-strain samples as in Figure 8. **: P < 0.01, *: P < 0.05, N = 3, compared ΔΔCT to non-strain and 0.5% strain groups.

To explore the effect of stretching magnitude on the ADSC responses to loading, we applied 0.5% (low), 2% (medium), and 10% (high) stretching during adipogenic induction in adult ADSCs and compared the results of Oil-Red staining and expression of adipogenic marker genes. As shown in Figure 9A, ADSCs that were subjected to 10% or 2% stretching presented fewer oil droplets than non-strained cells and the cells with 0.5% stretching. ADSCs subjected to medium or high strain also presented reduced AP2 and PPARγ expression (Figure 9B). Therefore, our results suggest that ADSC adipogenesis was reduced only by medium and high strain, but not by low strain.

## Discussion

The purpose of this study is to investigate the behavioral change of ADSCs in various degrees of aging with respect to self-renewal and differentiation induced by chemical cues. Another focus of this study is to explore improvement of the declined functions in aged ADSCs by mechanical loading. That ADSCs responded to 10% strain was previously reported [23,24], therefore, we used 10% strain in most experiments as the starting loading magnitude. We demonstrated that the result of mechanical loading in ADSCs depends on magnitude of the applied strain in which high strain (2-10%) significantly reduces adipogenesis and low strain (0.5%) has no effect (Figure 9). It should be noted that ADSCs in the mouse gonadal fat pad are not likely to experience 10% stretching *in vivo*, however, this magnitude of stretching is within the physiological range of loading for MSCs in muscular and peri-vascular tissues. We conducted our current study in ADSCs to take advantage of the ease of isolating large quantities of these cells from adult and aged animals. Further studies will be in need to verify that the observed results can be extrapolated to MSCs from other tissues as well as to the study of other forms of mechanical loading, using strain of various magnitudes, frequency, loading patterns, and durations.

Most current studies on MSCs have used established clones in which MSCs were selected through at least several rounds of passage and expansion. Aging (senescence) of MSCs in *in vitro *culture and loss of differentiation potential after the sixth passage has been demonstrated previously [25]. In this study, we chose to study primary ADSCs culture in very early passages (P0-P3) in order to explore functional differences in ADSCs of various ages in the absence of artifacts likely contributed by clonal selection and expansion in long-term cultures. SVF from adipose tissue is known to contain primary cells with high degree of heterogeneity. We found that more than 50% of P0 cells after *in vitro *culture for one week exhibited the CD34^-^/CD45^-^/CD105^+^/CD73^+^/CD90^+ ^surface markers, which are the most accepted surface markers for MSCs (data not shown). It should also be noted that > 95% of P6 young ADSCs were CD105^+^, CD73^+^, and CD90^+ ^(data not shown). We failed to obtain ADSC colonies beyond P10 from 21-month-old mice (data not shown), indicating that passage-related senescence increases rapidly in old ADSC culture.

Short-term mechanical loading that simultaneously activates many mitotic signaling pathways is a strong inducer for cell proliferation [26]. But, long-term mechanical loading (more than 24 h), which has not been as extensively studied as short-term loading, is less mitogenic [23,27]. We purposely investigated the cellular responses to long-term stretching (48 h) to distinguish the responses as the true adaptation of cells to mechanical loading from transient reactions to sudden changes in mechanical environment. Immediately following mechanical stretching, instead of an increase, a slight decrease (less than 5%) in the number of ADSCs was found (data not shown). Thus, it is possible, also as a limitation for similar membrane stretching systems that mechanical stretching selects for cells that firmly adhere to the elastic membrane and allows the detached cells to die of anoikis. As a result, firmly adhered cells would be enriched following mechanical loading. Because the cell loss after strain was not significant when compared to the whole population, we believe that anoikis does not play an important role in our result. Yet, cell adhesion to substrate is an inherent cellular property that may be associated with self-renewal and differentiation potential. Indeed, BMMSCs of various sizes and different morphologies have been shown to exhibit different adhesion property to substrate [28]. In a separate report, these heterogeneous cells also display different self-renewal abilities and express distinct surface markers [29]. In addition, the role of adhesion to substrate in dictating MSC differentiation potential was recently demonstrated [30]. Therefore, a change in ADSC adhesiveness to the substrate following mechanical loading should be investigated further. The finding that 48 h of pre-strain results in increased ADSC proliferation after replating (Figure 3) is intriguing to us. We did not find significant changes in the surface marker expression in ADSCs after applying strain (data not shown), therefore, the increase in proliferation following strain was not likely due to a selection for CD105^+^/CD73^+^/CD90^+ ^cells during mechanical loading.

Because of a clear increase of calcium deposition in pre-strained ADSCs as well as in young cells, we originally hypothesized that aging would reduce, and mechanical stretching would enhance the osteogenesis capacity of ADSCs. An increase in calcium deposition can result from an increase in the number of total colonies without increasing the percentage of contained osteoblast colonies, or from an increase in both total colony number and the ratio of contained osteogenic colonies. While the former only results from an increase in proliferation, the latter represents a true increase in osteogenic capacity. By using adult ADSCs as a model, we demonstrated that in pre-strained cells, calcium deposition, when normalized to the DNA content, was not increased by strain as compared to non-strained cells. This result corroborates with the real time PCR data, which showed that bone marker gene expression did not increase as a result of pre-strain. The conclusion that the osteogenic potential of ADSC colonies was not increased by pre-strain is analogous to the result that no loss of osteogenic potential occurred in old ADSCs. Our finding in murine ADSCs is in agreement with previous reports that the osteogenic potential of human ADSCs from old donors was not reduced [13,31]. In the literature, conflicting reports on the relative osteogenic potential of BMMSCs and ADSCs exist, and more findings indicate that ADSCs have an inferior osteogenic potential relative to BMMSCs [32-34]. Thus, it is also possible that we failed to detect changes in osteogenic potential due to aging or mechanical loading in ADSCs, while mechanical loading induces osteogenesis in BMMSCs in other reports, may result from the intrinsically lower osteogenic capacity of ADSCs.

The adipogenic program is regulated by multiple signaling pathways and involves the activation of numerous transcription factors. PPARγ serves as the pivotal transcription factor in adipogenesis. Several previous reports demonstrating that mechanical stretching inhibits PPARγ signaling in 3T3-L1 cells [35], BMMSCs [14] and skeletal myoblasts [36] may provide insight into the mechanism that underlies inhibition of adipogenesis by mechanical loading. We also found that a change in AP2 expression, previously considered a late marker of adipocytes, precedes the change in PPARγ expression induced in ADSCs by both aging and mechanical loading (10% strain). This result suggests that changes in PPARγ gene expression may not adequately reflect changes in PPARγ signaling, which can be better measured by the expression of its target genes. Indeed, AP2 is a PPARγ target gene [37,38]. We are currently examining the molecular basis of the inhibition of PPARγ signaling and reduction in adipogenesis as a result of mechanical strain. Two mechanical loading methods, pre-strain (PS) and simultaneous adipogenic induction and strain (AS), were compared in this study. It is not surprising to observe a stronger inhibitory effect of AS on adipogenesis, considering that the mechanical loading activates known signaling pathways leading to adipogenesis inhibition. We have found activation of non-canonical Wnt signaling by mechanical strain, involving calcium/calmodulin-dependent kinase II and nemo-like kinase, in various precursor cell lines and primary cells [39]. Therefore, inhibition of adipogenesis as a result of mechanical loading may be mediated by both canonical (β-catenin dependent) [21] and non-canonical Wnt pathways.

## Conclusions

Our results demonstrate an aging-dependent loss of self-renewal and increased propensity for adipogenesis in old ADSCs and a positive effect of mechanical loading that counteracts the aging factor. These findings are important for the further study on stem cell mechanobiology and reveal the benefits and potential of combining an *ex vivo *mechanical loading regime with autologous ADSC transplantation in treatment for a variety of aging related diseases.

## Methods

### Chemicals and culture medium

All chemicals used in this study were purchased from Sigma-Aldrich unless otherwise specified. All culture medium and reagents were purchased from Gibco-Invitrogen unless otherwise specified.

### Animals and ADSC isolation

All animal experiments were conducted in accordance with accepted standards of animal care and were approved by the Institutional Animal Care and Use Committee of the National Health Research Institutes in Taiwan. Male FVB/NarL mice were used in this study and were grouped into young (8-10 weeks), adult (5 months), and old (21 months) groups. After euthanization by CO_2_, gonadal fat pads were isolated and digested by following a published protocol with minor modifications [40]. In short, fat pieces were digested with 0.2% collagenase for 30 min at 37°C, followed by two, 5 min centrifugations at 260 × g to remove the adipocytes in supernatant. Following RBC lysis with 0.83% NH_4_Cl, the cells were washed twice with PBS, resuspended in maintenance medium, and plated in culture dishes for 4 h to remove non-adherent cells. The attached cells were considered SVF of P0 ADSCs. The isolated SVF was used immediately for experiments or cryopreserved for future studies. SVFs were cultured in maintenance medium consisting of αMEM supplemented with 10% fetal bovine serum and 1% penicillin-streptomycin at 37°C in a humidified with 5% CO_2_, and the medium was changed every 72 h.

### Application of mechanical strain

A Flexcell 4000T tensile system (Flexcell INT) with a 25 mm post was used to generate equibiaxial strain. 2.5 × 10^5 ^P2 ADSCs were plated in each well of a type 1 collagen-coated, flexible bottom plate (Flexwell) at 2.8 × 10^4 ^cells/cm^2 ^and cultured for another 16 h, followed by a change to fresh medium before loading to the strain system. The mechanical stretching was applied as sinusoidal wave of 0.5 Hz for 48 h with a peak strain of 0.5%, 2%, or 10% as indicated in the text. The medium in the plate of non-strain controls was also changed and placed in the same incubator as the strain samples. At the end of mechanical loading, ADSCs were collected by trypsin/EDTA, counted, and replated in 6-well plates for colony forming and differentiation assays. In studies with simultaneous mechanical strain and differentiation induction, cells were incubated with osteogenic or adipogenic medium at the beginning of the mechanical loading as described above, followed by a change to fresh differentiation medium at the end of loading and continued incubation as indicated in the text with medium changed every 72 h.

### Doubling time measurement and colony-forming assay

To measure the doubling time and colony-forming ability of ADSCs, 5000 cells were initially plated in each well of 6-well plates and cultured for various periods using maintenance medium. A cluster of cells consisting of at least five cells was considered a colony. The number of small (5-10 cells), medium (10-50 cells), and large (>50 cells) colonies formed by ADSCs in each well was counted.

### Osteogenic induction and Alizarin Red S or von Kossa staining

Osteogenic differentiation was induced using a previously described method [41]. In short, 2.5 × 10^4 ^ADSCs were seeded into each well of a 6-well plate, cultured for five days with maintenance medium, and then changed to differentiation induction medium. Osteogenic differentiation was induced by treating ADSCs with induction medium consisting of 100 nM dexamethasone, 10 mM sodium β-glycerophosphate, 0.05 mM L-ascorbic acid-2-phosphate (Fluka), and 10% fetal calf serum (FCS) in αMEM twice a week for 5, 14, and 21 days. For un-induced controls, cells were kept in maintenance medium. The degree of osteogenic differentiation was assessed by von Kossa or Alizarin Red S (ARS) staining for Ca^2+ ^deposition using previously described protocols [42,43]. After washing with calcium and phosphate-free saline, 70% ethanol-fixed cells were stained for 3 min with a 2% ARS solution (pH 4.2) at room temperature followed by washing with water and incubation with PBS for 15 min. To quantify the ARS staining result, the deposition was extracted by 10% (w/v) cetylpyridinium chloride in 10 mM sodium phosphate (pH 7.0) at room temperature for 1 h, and the ARS concentration in the extraction buffer was determined by measuring the absorbance at 562 nm. The absorbance values were normalized to the intensity of the ethidium bromide staining, which provides an estimation of the total DNA content of cells in a culture well. A standard curve correlating the intensity of ethidium bromide staining to the cell number in the well was established in parallel to correct for the factor used in normalization.

### Myogenic induction and Liu's stain

Myogenic differentiation was induced using a previously described protocol [44]. In short, sub-confluent cells were induced by 10 μM 5-azacytidine for 21 days. Myogenic differentiation was examined with Liu's stain for the appearance of multinucleated myotubes.

### Adipogenic induction and Oil-Red O stain

Adipogenic differentiation was induced as previously described [45] using induction medium consisting of 10 μM dexamethasone, 0.25 μM 3-isobutyl-1-methyl-xanthine, 4 μM recombinant human insulin, 10 μM troglitazone, and 10% FCS. Adipogenic differentiation was assayed by the formation of neutral lipid vacuoles stainable with Oil-Red O.

### Quantitative PCR for the measurement of osteogenic and adipogenic marker gene expression

Following osteogenic or adipogenic induction, ADSCs were lysed with Trizol to isolate RNA, followed by cDNA synthesis. Quantitative gene expression analysis was performed for mouse Runx2 and Bglap1 (*Osteocalcin, OC*) and normalized to GAPDH expression using Taqman gene expression assay (ABI system). The expression of adipogenic AP2 (*Fabp4*) and PPARγ was measured by Sybr Green-based real-time PCR, and the expression of β-actin was used as a control for constitutive expression. The sequences of probes and primers are listed in additional file 1.

### Statistical analyses

All experiments were performed at least in triplicate. The results shown are the mean values with error bars representing the SEM. The two-tailed, unpaired Student's t-test was used for analysis unless otherwise specified. A *P*-value < 0.05 was considered significant.

## Authors' contributions

SCH conceived the idea, performed the study, and applied for the grant supporting this research. TCW contributed significantly to this work, performed the study, and prepared the figures. HCY and MRC maintained the animal colonies and performed the study. CML and WSC performed the study. KML conceived the idea, applied for the grant supporting this research, and wrote the paper. All authors read and approved the final manuscript.

## Supplementary Material

Additional file 1 **Probes and primers used for quantitative PCR**. The sequences of Taqman probes and PCR primers used for quantitative PCR are listed in this file. Click here for file
