# Supplementary material for: Mechanical strain modulates age-related changes in the proliferation and differentiation of mouse adipose-derived stromal cells
Source: BMC Cell Biol. 2010 Mar 10;11:18. doi: 10.1186/1471-2121-11-18 (PMC2841110; doi:10.1186/1471-2121-11-18)
Supplement: Additional file 1 — Probes and primers used for quantitative PCR. The sequences of Taqman probes and PCR primers used for quantitative PCR are listed in this file. [file 1471-2121-11-18-S1.DOC]

The sequences of Taqman probes and PCR primers used for real-time PCR are listed as below.

|  | Taqman-FAM Probe sequence | PCR forward primer | PCR reverse primer |
| --- | --- | --- | --- |
| Runx2 | GGACGAGGCAAGAGTTTCACCTTGA | ABI assay ID_Mm00501580_m1 | |
| Bglap1 | CTGACCTCACAGATGCC | GGCTGCGCTCTGTCTCT | GACATGAAGGCTTTGTCAGACTCA |
| GAPDH | GAACGGATTTGGCCGTATTGGGCGC | ABI assay ID_Mm99999915_g1 | |

| Gene | Real time PCR forward primer | Real time PCR reverse primer |
| --- | --- | --- |
| AP2 | TTCGATGAAATCACCGCAGAC | GCTCATGCCCTTTCATAAACTC |
| PPARγ2 | AATCCTTGGCCCTCTGAGAT | TTTTCAAGGGTGCCAGTTTC |
| β-actin | ACACCCCAGCCATGTACG | TGGTGGTGAAGCTGTAGCC |
